# Longitudinal analysis of rotator cuff repair: joint kinematics and clinical outcomes

**DOI:** 10.1016/j.jseint.2025.101421

**Published:** 2025-12-02

**Authors:** Sujata Khandare, Rebekah L. Lawrence, Alena Jalics, Roger Zauel, Vasilios Moutzouros, Eric C. Makhni, Stephanie Muh, Michael J. Bey

**Affiliations:** a Department of Orthopaedic Surgery, Bone & Joint Center, Henry Ford Health, Detroit, MI, USA; b University of Michigan Transportation Research Institute, Ann Arbor, MI, USA; c Program in Physical Therapy, Washington University School of Medicine, St. Louis, MO, USA; d Department of Orthopaedic Surgery, Henry Ford Health, Detroit, MI, USA

**Keywords:** Rotator cuff, Joint kinematics, Strength, Patient-reported outcomes, Shoulder biomechanics, Tendon healing

## Abstract

**Background:**

Rotator cuff repair is a common surgical procedure, but postoperative outcomes can be highly variable, and postoperative repair tissue healing remains a significant clinical challenge. Furthermore, the biomechanical effects of rotator cuff repair are not fully understood.

**Methods:**

Twenty-two participants scheduled for arthroscopic rotator cuff repair were evaluated preoperatively and at 3, 12, and 24 months postsurgery. The following data were recorded at each time point: glenohumeral and scapulothoracic kinematics, shoulder strength, and patient-reported measures of pain and function. Postoperative repair integrity was assessed via magnetic resonance imaging at 3 months and 24 months postsurgery.

**Results:**

At 3 months postsurgery, 5 of 20 patients were identified as having a recurrent tear. An additional 4 patients had a recurrent tear at 24 months postsurgery. The center of contact of the humerus on the glenoid gradually shifted superiorly on the glenoid during the 24 month follow-up period (*P* < .01). There were subtle changes in scapulothoracic upward rotation at 3 months postsurgery compared to presurgery (*P* ≤ .01), but these differences did not persist at 12 or 24 months postsurgery. Compared to presurgery, there were significant increases in flexion and internal rotation strength at 12 months postsurgery (*P* < .01) and significant increases in flexion, abduction, external rotation, and internal rotation strength at 24 months postsurgery (*P* < .01). Patient-reported measures of pain and function improved significantly over the 24 month follow-up period (*P* < .01).

**Conclusion:**

Surgical rotator cuff repair was associated with decreased pain and improved subjective assessments of function within the first 3 months after surgery, and increased shoulder strength over 12- 24 months postsurgery. The study also found subtle changes over time in glenohumeral joint and scapulothoracic motion.

Rotator cuff tears are a common musculoskeletal condition, affecting approximately 40% of individuals over 60 and causing significant pain and functional impairment.^22^^,^^38^ In the United States alone, approximately 250,000 rotator cuff repairs are performed annually.^12^ Despite improvements over time in surgical repair technique and a greater than tenfold increase over the past 30 years in the number of rotator cuff articles published annually (PubMed), the postoperative healing process presents significant challenges with failure rates ranging from 20% to 70%.^6^^,^^11^^,^^13^^,^^14^^,^^31^^,^^35^ Furthermore, there remains significant variability in patient-reported outcomes following rotator cuff repair. For example, a recent meta-analysis by Holtedahl et al^19^ revealed such high variability across 46 prior studies as to preclude summary estimates of the effect of rotator cuff repair on patient-reported outcomes. Although research on the efficacy of rotator cuff repair has yielded consistency in some findings—eg, younger patients and those with smaller tears generally have better outcomes after rotator cuff repair^15^—the effects of rotator cuff repair on postoperative repair integrity, patient-reported outcomes, and their interaction are not yet fully understood.^27^

The specific biomechanical effects of rotator cuff repair are also not yet fully understood. For example, several prior studies have used motion capture analysis of skin-mounted markers to document glenohumeral joint (GHJ) or scapulothoracic kinematics after rotator cuff repair either longitudinally (ie, comparing to preoperative data) or in comparison to a separate control group.^17^^,^^23^^,^^33^^,^^39^ These studies have generally found that joint range of motion (ROM) after rotator cuff repair is often greater than the preoperative condition and generally increases over time.^23^^,^^33^ However, small differences or changes over time in joint motion must be interpreted with caution since the *in vivo* accuracy of motion capture systems that rely on skin-mounted markers is often unknown or not reported. To overcome limitations associated with skin-mounted markers, kinematic analysis of static or dynamic radiographic images has become increasingly common over the last 3nulldecades.^7^^,^^16^^,^^24^^,^^28^^,^^29^^,^^37^^,^^40^ The increased accuracy of these radiographic-based approaches has allowed for a more detailed assessment and understanding of shoulder kinematics, and yet the longitudinal progression of shoulder mechanics associated with the etiology and treatment of rotator cuff pathology is still not fully understood.

Prior work has demonstrated how rotator cuff pathology and physical therapy influence shoulder mechanics,^3^^,^^4^ and how shoulder mechanics change over time after surgical rotator cuff repair.^10^^,^^30^ However, it is important to note that none of these studies acquired data both before and after surgery in the same patients, and therefore the specific effects of surgical repair cannot be ascertained from these prior studies. Consequently, the objective of this study was to longitudinally assess the impact of surgical rotator cuff repair on shoulder kinematics, shoulder strength, and patient-reported outcomes. We accomplished this by measuring these outcomes at 4 critical time points: prior to surgery and at 3, 12, and 24 months postsurgery. The overarching hypothesis was that shoulder strength and patient-reported outcomes would improve after surgical rotator cuff repair, but that shoulder kinematics would not be restored to normal.

## Materials and methods

### Participants

In this study, 22 participants (16 male, 6 female, average age: 57.1 ± 5.6) between 50 and 70 years old were recruited and scheduled for arthroscopic repair of full-thickness rotator cuff tear. Potential participants were excluded if they had a body mass index greater than 32, had uncontrolled diabetes, were a current smoker, had sustained their rotator cuff tear through a traumatic injury, had prior shoulder surgery, had radiation exposure for other health-related reasons, or had received more than one corticosteroid injection. Informed consent was obtained from each patient before participation and ethical approval was obtained from the Henry Ford Health Institutional Review Board (IRB No.: 12,146; Board Name: HFH Institutional Review Board-Henry Board-IRB00000253).

All rotator cuff repairs were performed by one of 3 fellowship-trained orthopedic surgeons and involved debriding the supraspinatus tendon footprint and using suture anchors to reattach the retracted tendon to its anatomical insertion on the greater tuberosity. The choice of repair technique, including the number of rows and anchors used, was left to the surgeon's discretion. Although variability in approach and experience may be confounding factors, it helps to ensure the generalizability of outcomes.

### Physical therapy

Postsurgery, patients were provided with postoperative medications along with a shoulder abduction sling and necessary precautions. They were also enrolled in a physical therapy program, which included: (1) only ROM exercises for the initial 6 weeks postsurgery, (2) a transition to active-assisted ROM exercises after the sixth postoperative week, (3) a progression to active ROM exercises by the eighth postoperative week, (4) the introduction of isometric strengthening exercises between the sixth and eighth weeks postsurgery, and (5) a progression to resisted exercises after the 10th-12th postoperative weeks. The progression of these rehabilitation exercises was tailored to each patient's tolerance level and focused on preventing compensatory movement patterns, such as shoulder shrugging.

### Data collection

At the 4 critical time points, *in vivo* shoulder kinematics were measured using a biplanar videoradiographic system comprising of 2 high-voltage pulsed X-ray generators (model CPX 3100CV; EMD Technologies), 2 40-cm image intensifiers (model P9447H110; Thales), and 2 cameras (Phantom VEO 340; Vision Research).^3^^,^^4^^,^^9^ During imaging, patients were seated with their involved shoulder positioned centrally within the 3D imaging volume and protected by a lead apron. Images were acquired while the patients performed scapular plane humeral abduction through their pain-free existing ROM, starting with their arm at their side. Patients were instructed to perform this motion in 2 s and practiced the motion trial prior to image acquisition to ensure the proper motion plane and pacing. Three trials were acquired, with a minimum of 2 min between trials. In addition, images were acquired during one additional static trial with the patient's arm resting passively at their side.

A computed tomography scan of the humerus, scapula, and upper thorax was also acquired from each patient. The computed tomography images were manually segmented using Mimics (Materialise NV, Leuven, Belgium), and 3D bone models for the humerus, scapula, and ribs 3 and 4 were developed. The 3D locations of anatomical landmarks were identified and used to define anatomical coordinate systems for the humerus, scapula, and thorax.^8^, ^9^, ^10^ The 3D position and orientation of the humerus, scapula, and ribs 3 and 4 were measured from the biplane videoradiographic images to determine conventional glenohumeral, humerothoracic, and scapulothoracic kinematics (internal rotation, upward rotation, and tilt).^4^^,^^9^ Lawrence et al^25^ also provides detailed description of the approach used to acquire images, measure shoulder motion, develop 3D bone models, track the position and orientation of the humerus, scapula, and torso, and calculate kinematic outcome measures.

To potentially identify issues like altered movement patterns or abnormal joint loading, GHJ contact patterns were estimated by integrating joint motion data, obtained from biplane videoradiographic images, with patient-specific bone models.^8^ The process involved estimating the GHJ contact center for each data frame by calculating the centroid of the shortest distance between the surfaces of the humerus and glenoid bone models. This contact-center position was then expressed relative to the glenoid coordinate system. To accommodate variations in patient size, these GHJ contact center estimates were normalized based on the glenoid height and width, as measured on the patient-specific bone models. Using this information, the average anteroposterior and superoinferior contact-center position for each trial was calculated. The range of anteroposterior and superoinferior contact-center and the length of the contact-center path were also calculated. Using this approach for estimating GHJ contact patterns, acromiohumeral distance (AHD) was determined by calculating the shortest distance between the humerus and acromion surfaces for every data frame and computing the average distance across the entire trial. During the dynamic task, there were differences in glenohumeral ROM across patients and within a patient across the 4 time points. To account for this variability, a common ROM across all 4 time points was calculated for each patient, and GHJ contact-center and AHD data were determined for this common range of glenohumeral elevation.

To track the progression of functional recovery after rotator cuff repair, shoulder function was assessed at the 4 critical time points. Isometric shoulder strength was assessed using an isokinetic dynamometer (Biodex System 2; Biodex Medical Systems, Shirley, NY, USA) using methods previously described.^10^ Strength measurements were conducted during frontal-plane abduction at 30°, sagittal-plane flexion at 30° elevation, internal rotation at 15° of frontal-plane abduction with the humerus in a neutral rotation position, and external rotation at 15° of frontal-plane abduction with the humerus in a neutral rotation. Strength was normalized against normative values based on characteristic variables like age, sex, body mass, and side dominance.^20^ Patient-reported pain and function were assessed using a 10-cm visual analog scale and the Western Ontario Rotator Cuff Index (WORC), respectively.

In addition, postoperative repair integrity was assessed via magnetic resonance imaging (MRI) at 3 and 24 months postsurgery. MRI images were reviewed by musculoskeletal radiologists according to the 5 categories described by Sugaya et al.^34^ Repairs classified as type IV (minor discontinuity in only 1 or 2 slices) or type V (major discontinuity in more than 2 slices) were considered recurrent tendon defects.

### Statistical analysis

Patient characteristic data were summarized using mean values and standard deviations or proportions, as applicable. Changes in strength, ROM, visual analog scale, and WORC scores over time were assessed using a one-factor repeated measures analysis of variance. Changes in kinematic outcome measures were assessed throughout the ROM (glenohumeral elevation angles: 20°, 40°, 60°) and over time (presurgery, 3, 12, and 24 months postsurgery) were analyzed using a two-factor repeated measures analysis of variance. All follow-up tests were made using Tukey post hoc adjustments. For the two-factor model, main effects were only interpreted in the absence of a two-factor interaction. Significance was set at *P* ≤ .05.

## Results

### Sample sizes

Recruitment and retention of participants for this project were significantly impacted by the COVID-19 pandemic. Specifically, 22 patients were recruited for the preoperative assessment, but only 20 patients were willing and/or able to be tested at 3 months postsurgery, 16 patients at 12 months postsurgery, and 12 patients at 24 months postsurgery.

### Rotator cuff repair integrity

At 3 months postsurgery, MRI data were available for 20 patients. Of those 20 patients, 5 (25%) were identified as having a recurrent tear (ie, failed repair). At 24 months postsurgery, MRI data were available for 12 patients. Of those 12 patients, 5 were identified as having a recurrent tear. These 5 patients consisted of 1 patient who had a tear at 3 months postsurgery and 4 patients who had a retear between 3 and 24 months postsurgery.

### Glenohumeral and scapulothoracic kinematics

**Figure 1:**
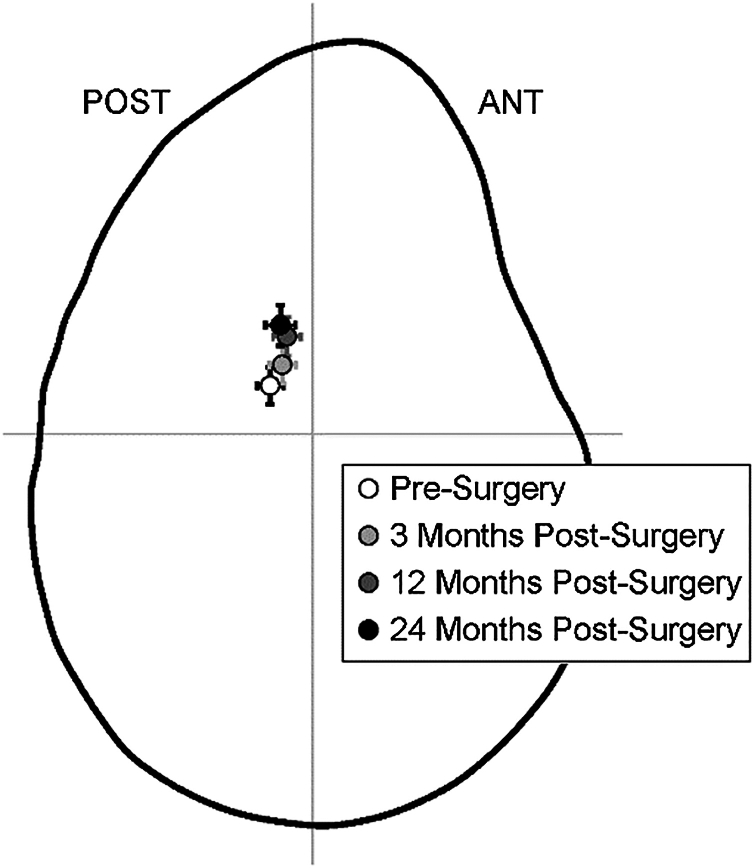
Mean (± standard error) glenohumeral joint contact center shown on a lateral view of the glenoid. The position of the contact center shifted superiorly over time (*P* < .01), but no change in ANT/ POST position of the contact center was detected (*P* = .61). *ANT/POST*, anterior/posterior.

The normalized glenohumeral superior/inferior (S/I) contact center location gradually shifted more superiorly during the 24 month follow-up period (main effect: *P* < .01, Table I, Fig. 1). At the presurgery visit, the humerus was located an average ± standard error of 6.2% ± 2.4% superior to the glenoid center, and this increased to 12.4% ± 2.5% at 12 months postsurgery (*P* = .01) and to 13.9% ± 2.6% at 24 months postsurgery (*P* < .01). Statistically significant changes over 24 months postsurgery were not observed for normalized glenohumeral anterior/posterior (A/P) contact center location (*P* = .61, Table I), normalized contact path length (*P* = .90, Table I), or AHD (*P* = .12, Table I). Under static conditions, statistically significant changes over 24 months postsurgery were not detected for the normalized GHJ A/P (*P* = .83) or S/I) (*P* = .44) contact center locations (Table II).

**Table I:** Average (± standard error) GHJ kinematics during scapular-plane abduction.

Outcome measure (units)	Presurgery	Months postsurgery		
		3	12	24
GHJ contact center, A/P (% glenoid width)	−7.6 (2.4)	−5.4 (2.3)	−4.6 (2.5)	−5.8 (2.7)
GHJ contact center, S/I (% glenoid height)	6.2 (2.4)	8.8 (2.3)	12.4 (2.5)	13.9 (2.6)
GHJ contact path length (% glenoid height)	41.7 (6.6)	45.5 (6.4)	44.9 (6.9)	38.3 (7.2)
acromiohumeral distance (mm)	2.9 (0.3)	2.7 (0.3)	2.4 (0.3)	2.6 (0.3)

*A/P*, anterior/posterior; *S/I*, superior/inferior; *GHJ*, glenohumeral joint.

**Table II:** Average (± standard error) GHJ kinematics during static conditions (arm at side).

Outcome measure (units)	Presurgery	Months postsurgery		
		3	12	24
GHJ contact center, A/P (% glenoid width)	−3.6 (3.3)	−1.5 (3.9)	1.1 (3.3)	−0.6 (1.0)
GHJ contact center, S/I (% glenoid height)	−10.2 (4.6)	−17.9 (3.4)	−10.5 (4.6)	−15.2 (3.5)

*A/P*, anterior/posterior; *S/I*, superior/inferior; *GHJ*, glenohumeral joint.

**Figure 2:**
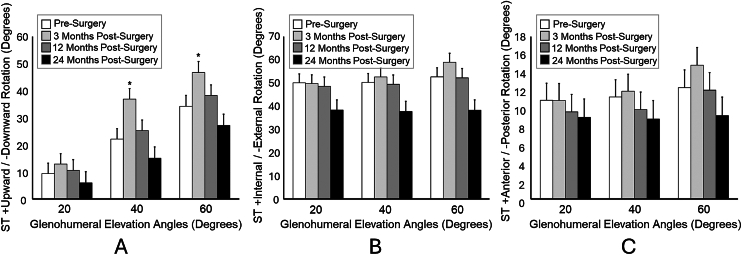
(**A**) Compared to presurgery, ST upward rotation was significantly greater at 3 months postsurgery only at 40° and 60° of glenohumeral elevation. ∗*P* < .05. (**B**) Differences across the study period also existed for scapulothoracic internal rotation (time-by-angle interaction: *P* = .02; however, pairwise follow-ups failed to meet statistical significance (*P* > .06). (**C**) No differences were observed for scapulothoracic tilt (time-by-angle interaction: *P* = .17, time main effect: 0.16. *ST*, scapulothoracic.

Scapulothoracic upward rotation kinematics changed during the 24-month study period and depended on the angle of glenohumeral elevation (time-by-angle interaction: *P* < .01). Specifically, patients were in an average of 12.5°-14.8° more upward rotation at 40° and 60° glenohumeral elevation at 3 months postsurgery compared to presurgery (*P* ≤ .01, Fig. 2*A*). However, upward rotation decreased by the 12-month and 24-month visits such that neither time points were statistically different from presurgery (*P* ≥ .22, Fig. 2*A*). Differences across the study period also existed for scapulothoracic internal rotation (time-by-angle interaction: *P* = .02, Fig. 2*B*); however, pairwise follow-ups failed to meet statistical significance (*P* ≥ .06). Finally, no differences were observed for scapulothoracic tilt (time-by-angle interaction: *P* = .17, time main effect: 0.16, Fig. 2*C*).

### Shoulder strength

**Figure 3:**
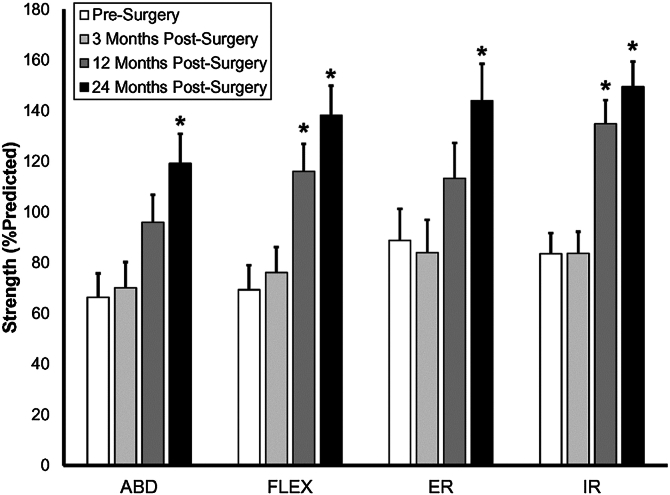
Compared to presurgery, shoulder strength increased significantly for FLEX and IR at 12 months postsurgery, and for all tested motions at 24 months postsurgery. *ABD*, abduction; *ER*, external rotation; *IR*, internal rotation; *FLEX*, flexion. ∗*P* < .05.

Significant increases in strength were not observed for any motion between baseline and 3 months postsurgery (*P* ≥ .90, Table III, Fig. 3). However, compared to baseline, significant increases were observed at 12 months postsurgery in both normalized flexion strength (46.7% ± 10.7%, *P* < .01) and normalized internal rotation strength (51.3% ± 9.1%, *P* < .01). By 24 months postsurgery, increases were observed in all motions (abduction: 52.8% ± 12.5%, *P* < .01; flexion: 68.9% ± 11.5%, *P* < .01; external rotation: 55.2% ± 12.9%, *P* < .01; internal rotation: 66.0% ± 9.6%; *P* < .01).

**Table III:** Average (± standard error) shoulder strength, VAS pain score, and WORC score.

Outcome measure (units)	Presurgery	Months postsurgery		
		3	12	24
ABD strength (% predicted)	66.4 (9.4)	70.2 (10.0)	96.0 (10.7)	119.1 (11.7)
FLEX strength (% predicted)	69.3 (9.7)	76.1 (10.0)	116.1 (10.8)	138.2 (11.6)
ER strength (% predicted)	88.7 (12.5)	84.0 (12.9)	113.3 (14.0)	143.9 (14.5)
IR strength (% predicted)	83.5 (8.2)	83.7 (8.5)	134.8 (9.3)	149.6 (9.8)
VAS pain (scale: 0 to 10)	7.8 (0.5)	4.6 (0.5)	1.9 (0.5)	1.4 (0.6)
WORC (scale: 0 to 100%)	41.1 (4.1)	60.8 (4.3)	83.4 (4.7)	90.3 (5.0)

*ABD*, abduction; *FLEX*, flexion; *ER*, external rotation; *IR*, internal rotation; *VAS*, visual analog scale; *WORC*, Western Ontario Rotator Cuff Index.

### Patient-Reported Outcome Measures (PROMs)

Patient-reported pain decreased consistently over the duration of the study (*P* < .01, Table III). Prior to surgery, patients reported an average pain rating of 7.7 ± 0.5. This decreased to 4.6 ± 0.5 at 3 months postsurgery (*P* < .01), 1.9 ± 0.5 at 12 months postsurgery (*P* < .01), and 1.4 ± 0.6 at 24 months postsurgery (*P* < .01). Likewise, patient-reported function (WORC) also increased over the study period (*P* < .01, Table III). Prior to surgery, patients reported an average WORC score of 41.1% ± 4.1%. This increased to 60.1% ± 4.3% at 3 months postsurgery (*P* < .01), 83.4% ± 4.7% at 12 months postsurgery (*P* < .01), and 90.3% ± 5.0% at 24 months postsurgery (*P* < .01).

## Discussion

This longitudinal study aimed to comprehensively analyze the progression of biomechanical and patient-reported outcomes associated with rotator cuff repair. The study demonstrated that the center of contact of the humerus on the glenoid during shoulder abduction shifted superiorly on the glenoid over time, while the early changes in scapulothoracic motion after surgical repair did not persist over time. Shoulder strength improved, pain decreased, and patient-reported outcomes increased significantly over time after surgery.

**Figure 4:**
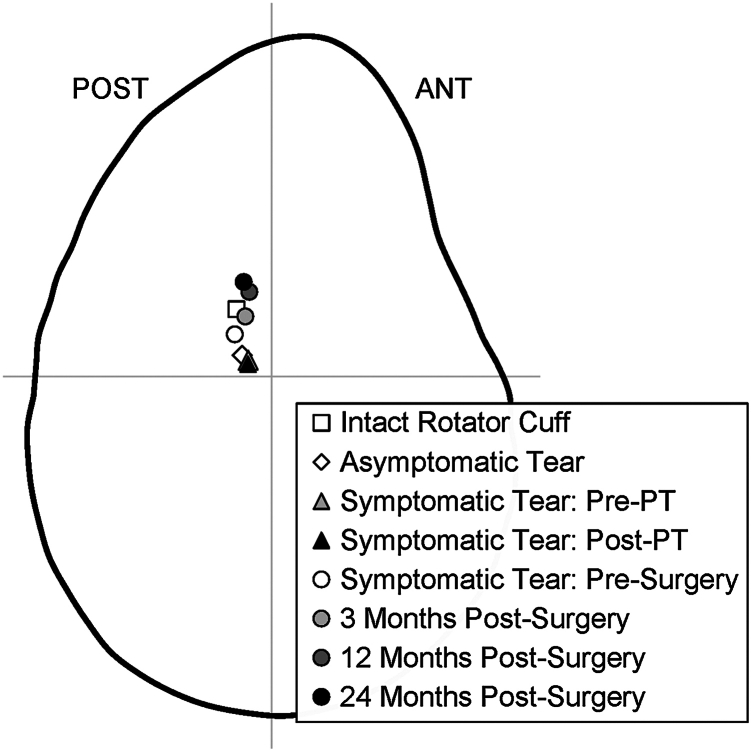
Progression of the glenohumeral joint contact center from an intact rotator cuff through 24 months postsurgery. This figure includes data (the noncircular markers) reproduced from Baumer et al.^2^^,^^3^*ANT*, anterior; *POST*, posterior; *PT*, physical therapy.

When interpreted in conjunction with prior studies by Baumer et al,^3^^,^^4^ the findings from this study provide a more comprehensive understanding of how GHJ mechanics change with the progression from a healthy shoulder (ie, intact rotator cuff) through 2 years postsurgery. Briefly, the GHJ contact center: (1) is located roughly 10% of the glenoid height above the glenoid's midline in individuals with an intact rotator cuff, (2) moves inferiorly on the glenoid with asymptomatic rotator cuff pathology, (3) moves farther inferiorly on the glenoid with symptomatic rotator cuff pathology, (4) remains unchanged as a result of physical therapy, (5) is located more superiorly on the glenoid prior to surgical repair, and (6) continues to move superiorly on the glenoid over time postsurgery (Fig. 4). In a healthy shoulder, coordinated activation of the rotator cuff and deltoid muscles forms balanced force couples that stabilize the GHJ and prevent proximal migration of the humeral head. In the coronal plane, the deltoid's upward force is countered by the compressive action of the rotator cuff, which maintains joint congruency through the concavity-compression mechanism. Disruption of this balance reduces this stabilizing effect and allows superior translation of the humeral head, indicating loss of the normal force couple and potential compromise of joint stability.^2^

Although these data were not acquired in the same patients and should be interpreted with caution, they raise new questions. For example, it is unclear why the GHJ contact center prior to surgery is located higher on the glenoid than the post–physical therapy (PT) condition (Fig. 4). It is plausible that the higher contact center in presurgical condition indicates a level of rotator cuff pathology unresponsive to PT and may be associated with pain prompting surgery. However, Baumer et al^3^ reported that PT significantly decreased pain despite no change in contact center, whereas the current study reports that surgical repair also significantly decreased pain but was accompanied by significant changes in GHJ contact center over time. Taken together, these findings suggest that pain that is severe enough to warrant surgical intervention may have little to do with GHJ mechanics, though their complex relationship warrants further investigation.

Another question raised by this study is why the GHJ contact center continued to migrate superiorly on the glenoid over time postsurgery (Figs. 3 and 4). One potential contributing factor may be differences in postoperative repair integrity, as MRI data indicated that several patients experienced recurrent tears between 3 and 24 months postsurgery. Superior migration of the contact center in these cases may reflect incomplete restoration of rotator cuff force couple. Although decreased adherence to postoperative rehabilitation may also play a role.

Patients were enrolled in a postoperative rehabilitation program based on the American Society of Shoulder and Elbow Therapists' consensus statement,^36^ completing an average (±standard deviation) of 19.6 ± 7.9 (range: 2-31) supervised rehabilitation visits over 15 ± 5.3 (range: 5-24) weeks postsurgery. However, adherence to rotator cuff–specific strengthening exercises likely declined over the 24-month period, which may have contributed to reduced dynamic GHJ stability. This explanation remains speculative, as home-based rehabilitation was not documented, and other factors, such as altered motor control or changes in muscle-tendon mechanics due to repair tissue elongation,^26^ may also have influenced this finding.

The contact center path length did not change significantly over time (*P* = .90, Table I). Although the superior shift in the S/I contact center might suggest that the resting position of the humerus on the glenoid is also shifting superiorly over time. However, data acquired under static conditions does not support this interpretation. Rather, it indicates that the humerus sits substantially lower on the glenoid, slightly posterior and inferior to the center of the glenoid coordinate system at all time points, with no change in the S/I (*P* = .44) or A/P (*P* = .83) resting position over time (Table II). These findings suggest that the superior shift may reflect altered loading patterns or adaptive kinematic behavior or loss of dynamic GHJ stability. However, given improvements in strength, WORC scores, and pain, any functional relevance of these subtle kinematic changes or progressive loss of dynamic GHJ stability are unclear.

Changes in average AHD were not detected over time (*P* = .12, Table III). This finding is consistent with previous work indicating minimal change in AHD over 12 months postsurgery in patients with intact rotator cuff repairs.^21^ Despite the superior shift of the GHJ contact center, no corresponding decrease in AHD was observed. Since the study had incomplete follow-up due to unanticipated attrition and COVID-19 pandemic-related testing restrictions, comparisons between dissimilar patient populations may have masked significant changes in AHD over time. However, a secondary analysis of 11 patients with complete follow-up showed only a slight, nonsignificant (*P* = .77) decrease in AHD over time (4.2 ± 1.3 mm presurgery, 3.9 ± 1.0 mm at 3 months, 3.7 ± 0.9 mm at 12 months, and 3.8 ± 0.9 mm at 24 months postsurgery. In the absence of a technical explanation, subtle (albeit not significant) changes in scapular posterior tilt (Fig. 2*C*) may have compensated for the humerus being positioned more superiorly on the glenoid during motion. This adaptation may have preserved AHD and minimized changes over time. Although speculative, this explanation suggests a complex interaction between glenohumeral (ie, AHD) and scapulothoracic motion that remains not well understood.

Shoulder strength (Fig. 3) and subjective assessments of pain and function (Table III) improved over time after rotator cuff repair, consistent with previous studies on strength,^32^ pain,^18^ and patient-reported outcomes^1^^,^^5^ after cuff repair. From presurgery to 3 months postsurgery pain scores decreased by roughly 40% and WORC scores improved by 20%, but there was no significant change in shoulder strength (Fig. 3). These results indicate that patients can expect reduced pain and improved shoulder function as the most immediate effects of rotator cuff repair, while full restoration of shoulder strength may take a year or 2 postsurgery.

Although measuring maximum strength is a convenient method to assess shoulder function in both laboratory and clinical setting, it may have little relevance to activities of daily living. This is because most activities of daily living require only a small fraction of maximum strength, and few individuals regularly perform activities that challenge their maximum strength. Accordingly, studies investigating associations between maximum strength and joint mechanics (eg, contact center location) during activities with low strength requirements have found few significant associations.^10^^,^^30^ Future research should prioritize strength assessments that better reflect functional capacity in activities of daily living, such as measures of muscular endurance at low force levels, to more accurately evaluate shoulder function recovery after rotator cuff repair.

As with any study, findings reported here should be interpreted in light of certain limitations. The most significant was attrition and incomplete follow-up due to the COVID-19 pandemic. Every effort was made to accommodate patients and maintain the testing schedule, but institutional restrictions during the first two years of the pandemic severely limited in-person testing. Many patients were understandably hesitant to return for in-person testing after restrictions were lifted and instead elected to withdraw from the study. Consequently, the sample size was small and unbalanced across time points, which unfortunately precluded subgroup analyses (eg, intact vs. retear). In addition, the absence of a nonoperative control group limits direct comparison to normative kinematic behavior and should be addressed in future studies. Nevertheless, the longitudinal study design and rigorous experimental methods enabled detection of significant changes in GHJ motion, scapulothoracic motion, shoulder strength, and subjective assessments of pain and function over time.

## Conclusion

This study demonstrated that rotator cuff repair is associated with decreased pain and improved subjective assessments of shoulder function within the first 3 months after surgery, and increased shoulder strength over 12-24 months postsurgery. The study also indicated subtle changes over time in GHJ and scapulothoracic motion, but the functional implications of these biomechanical changes remain unclear. A more mechanistic understanding of the factors impacting GHJ and scapulothoracic motion, along with a more rigorous assessment of the functional and potential long-term implications of subtle changes in joint motion, is a potential fruitful avenue of future research.
